# PEST-containing nuclear protein regulates cell proliferation, migration, and invasion in lung adenocarcinoma

**DOI:** 10.1038/s41389-019-0132-4

**Published:** 2019-03-14

**Authors:** Da-Yong Wang, Ya Hong, Ya-Ge Chen, Peng-Zhen Dong, Shi-Yu Liu, Ying-Ran Gao, Dan Lu, Hui-Min Li, Tao Li, Jian-Cheng Guo, Fei He, Xue-Qun Ren, Shi-Yong Sun, Dong-Dong Wu, Shao-Feng Duan, Xin-Ying Ji

**Affiliations:** 1 The First Affiliated Hospital of Henan University, Kaifeng, Henan 475001 China; 2 School of Basic Medical Sciences, Henan University College of Medicine, Kaifeng, Henan 475004 China; 3 Joint National Laboratory for Antibody Drug Engineering, Henan International Joint Laboratory for Nuclear Protein Regulation, Henan University, Kaifeng, Henan 475004 China; 4 Center for Precision Medicine, Zhengzhou University, Zhengzhou, Henan 450052 China; 5 Huaihe Hospital of Henan University, Kaifeng, Henan 475000 China; 6 Department of Hematology and Medical Oncology, Emory University School of Medicine and Winship Cancer Institute, Atlanta, GA 30322 USA; 7 College of Pharmacy, Henan University, Kaifeng, Henan 475004 China

## Abstract

Lung cancer is the leading cause of cancer-related mortality worldwide. PEST-containing nuclear protein (PCNP) has been found in the nucleus of cancer cells. Whether PCNP plays a role in the growth of lung adenocarcinoma is still unknown. In the present study, the results indicated that the level of PCNP in lung adenocarcinoma tissue was significantly higher than that in corresponding adjacent non-tumor tissue. Over-expression of PCNP promoted the proliferation, migration, and invasion of lung adenocarcinoma cells, while down-regulation of PCNP exhibited opposite effects. PCNP over-expression decreased apoptosis through up-regulating the expression levels of phospho (p)-signal transducers and activators of transcription (STAT) 3 and p-STAT5 in lung adenocarcinoma cells, whereas PCNP knockdown showed opposite trends. PCNP overexpression enhanced autophagy by increasing the expression levels of p-phosphatidylinositol 3-kinase (PI3K), p-Akt, and p-mammalian target of rapamycin (mTOR) in lung adenocarcinoma cells, however an opposite trend was observed in the sh-PCNP group. In addition, overexpression of PCNP showed the tumor-promoting effect on xenografted lung adenocarcinoma, while PCNP knockdown reduced the growth of lung adenocarcinoma via regulating angiogenesis. Our study elucidates that PCNP can regulate the procession of human lung adenocarcinoma cells via STAT3/5 and PI3K/Akt/mTOR signaling pathways. PCNP may be considered as a promising biomarker for the diagnosis and prognosis in patients with lung adenocarcinoma. Furthermore, PCNP can be a novel therapeutic target and potent PCNP inhibitors can be designed and developed in the treatment of lung adenocarcinoma.

## Introduction

Lung cancer is the leading cause of cancer-related death in the world^1,2^. Lung cancer can be divided into many histological categories, including lung adenocarcinoma, large cell carcinoma, squamous cell lung carcinoma, and small cell lung carcinoma^3^. The majority of patients with lung cancer present with locally advanced/metastatic disease, which will lead to a poor prognosis^4^. The 5-year overall survival rate of patients with advanced lung cancer or metastatic lung cancer remains less than 20%^5^. Immune checkpoint therapy, particularly anti-programmed cell death receptor-1 (PD-1)/anti-programmed cell death ligand-1 (PD-L1) antibody, is a novel cancer therapy and has become the standard therapy for a variety of tumors, including non-small cell lung cancer (NSCLC)^6–8^. Nevertheless, the clinical benefit is limited to a subset of patients, which can be attributed to immunosuppressive tumor microenvironments and individual differences in tumor immunogenicity^6,9^. Oncogenic mutations in the epidermal growth factor receptor (EGFR) tyrosine kinase domain have been found in NSCLC^10,11^. EGFR tyrosine kinase inhibitors (TKIs) are regarded as the standard first-line treatment of patients with advanced/recurrent NSCLC harboring activating EGFR mutations^10,12,13^. However, patients treated with EGFR-TKIs can develop resistance against these drugs^10,12^. Therefore, identification of specific molecular targets and development of effective therapeutic strategies are still urgently needed for the treatment of lung cancer^2,4,14^.

PEST is a peptide sequence which is rich in proline (P), glutamic acid (E), serine (S), and threonine (T)^15–17^. PEST-containing nuclear protein (PCNP) is firstly identified in the nucleus by database mining^18^. Recent studies indicate that PCNP mRNA has been detected in several cancer cells, including HepG2 hepatoma cells, U-937 myeloid leukemia cells, and HT-1080 fibrosarcoma cells, suggesting that PCNP may be involved in some aspects of tumorigenesis^18,19^. Our previous study has shown that PCNP could mediate the growth of human neuroblastoma cells via mitogen-activated protein kinase (MAPK) and phosphatidylinositol 3-kinase (PI3K)/Akt/mammalian target of rapamycin (mTOR) pathways^20^. However, the expression level of PCNP in lung adenocarcinoma remains unknown, as well as the mechanism of action of PCNP on the procession of lung adenocarcinoma has not yet been elucidated.

In the current study, the expression level of PCNP in human lung adenocarcinoma was examined. The mechanism of action of PCNP in the proliferation, migration, and invasion of human lung adenocarcinoma cells was investigated. The effects of PCNP on tumor growth and angiogenesis in nude mice bearing with human lung adenocarcinoma were further determined.

## Results

### PCNP protein level is higher in human lung adenocarcinoma tissue than that in adjacent normal tissue

**Fig. 1:**
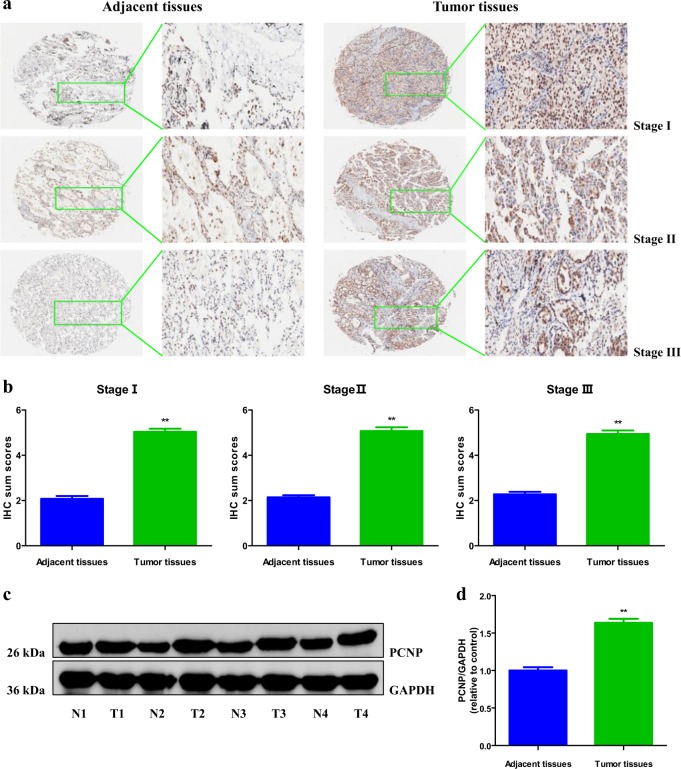
The expression of PCNP in human lung adenocarcinoma tissues. **a** IHC results of PCNP expression in different clinical stages of human lung adenocarcinoma tissues and adjacent tissues (left: ×400; right: enlarged). **b** IHC sum scores were adopted to compare PCNP expression in different clinical stages of human lung adenocarcinoma tissues and adjacent tissues. **c** Representative results of PCNP protein expression in fresh human lung adenocarcinoma tissues (T) and adjacent normal tissues (N) detected by Western blotting. Glyceraldehyde-3-phosphate dehydrogenase (GAPDH) was used as the loading control. **d** Statistical results showed that the protein levels of PCNP were significantly elevated in fresh human lung adenocarcinoma tissues compared to adjacent normal tissues. Data are presented as mean ± standard error of the mean (SEM). **P < 0.01 compared with adjacent normal tissues

In light of the fact that lung adenocarcinoma is the major form of lung cancer, lung adenocarcinoma was investigated in the present study. In order to determine the level of PCNP in human lung adenocarcinoma tissue, we examined PCNP level in human lung adenocarcinoma tissue chip that includes 63 lung adenocarcinoma specimens and adjacent non-tumor tissues by immunohistochemistry (IHC). Our results indicated that the level of PCNP was higher in all clinical stages of human lung adenocarcinoma than that in adjacent tissues (Fig. 1a, b). We further determined the level of PCNP in fresh surgical specimens of lung adenocarcinoma and corresponding adjacent normal tissues. The results were in line with the conclusions mentioned above that PCNP level was high in lung adenocarcinoma tissues but low in adjacent non-tumor tissues (Fig. 1c, d). To determine the clinical significance of PCNP in lung adenocarcinoma, we further analyzed the association of PCNP level with clinicopathological parameters in lung adenocarcinoma tissue chip (Table 1). The results showed that PCNP level was associated with T classification of lung adenocarcinoma. Overall, the results indicate that PCNP may be a promising biomarker for diagnosis and prognosis of lung adenocarcinoma and can serve as a growth regulator in lung adenocarcinoma cells.

**Table 1:** Association between PCNP expression and clinicopathological characteristics of patients with lung adenocarcinoma (*n* = 63)

Characteristics	Cases	PCNP expression		*P* value
		Low	High	
Age (years)				0.195
≤65	47	20	27	
>65	16	4	12	
Gender				0.667
Male	33	15	18	
Female	30	9	21	
Tumor size (cm)				0.147
≤5	50	18	32	
>5	13	6	7	
Disease grade				0.12
I	7	4	3	
II	42	17	25	
III	14	3	11	
T classification				0.035
T1	19	6	13	
T2	32	15	17	
T3	10	2	8	
T4	2	1	1	
Lymph node status				0.969
N_X_、N0	45	16	29	
N1、N2、N3	18	8	10	

### PCNP mediates the proliferation and viability of human lung adenocarcinoma cells

**Fig. 2:**
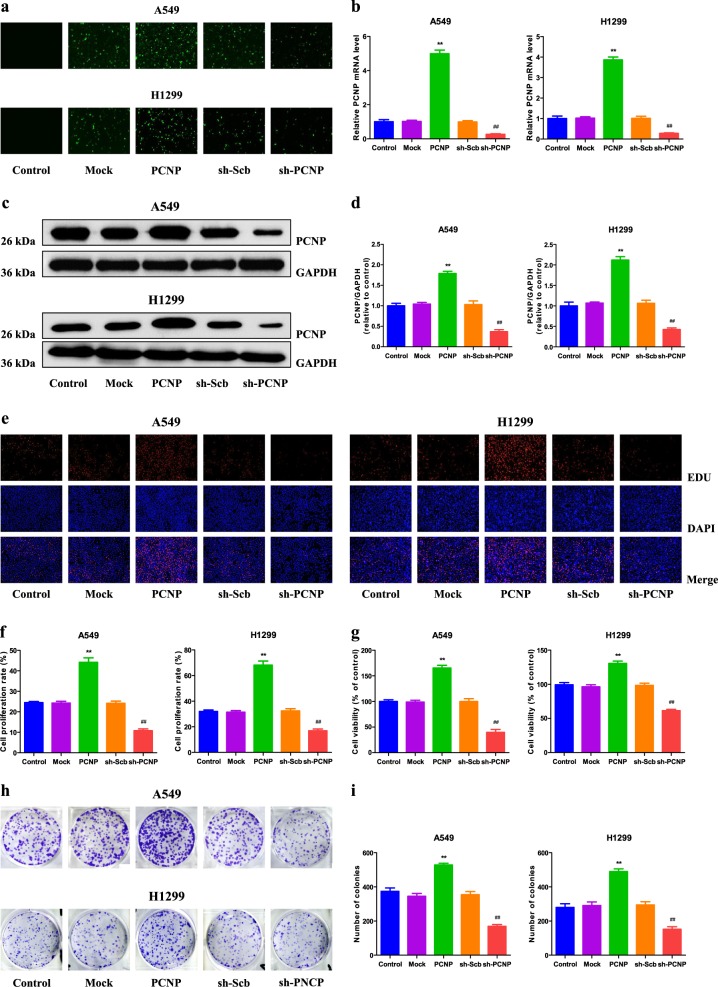
Effects of PCNP on the proliferation and viability of human lung adenocarcinoma cells. **a** Fluorescence microscopy of PCNP in A549 and H1299 cells; original magnification ×100. **b** The expression level of PCNP mRNA was examined by reverse transcription-polymerase chain reaction (RT-PCR). The results were normalized to the level of GAPDH. **c** The protein expression of PCNP was examined by Western blotting. GAPDH was used as the loading control. **d** The densitometry analysis of PCNP was performed, normalized to the corresponding GAPDH level. **e** DNA replication activities of A549 and H1299 cells in each group were examined by 5-ethynyl-2’-deoxyuridine (EdU) assay; original magnification ×100. **f** The proliferation rate of each group was analyzed. **g** The percentages of viable cells were determined using CellTiter 96 AQ_ueous_ One Solution Cell Proliferation Assay (MTS) and the cell viability of the control group was taken as 100%. **h** The clonogenic capacity was determined in A549 and H1299 cells. **i** The numbers of colonies larger than 0.1 mm in diameter were counted. Data are presented as mean ± SEM of three independent experiments; **P < 0.01 compared with the Mock group; ^##^*P* < 0.01 compared with the sh-Scb group

To detect the role of PCNP in the growth of human lung adenocarcinoma cells, overexpression and knockdown experiments were performed. Transfection of PCNP into A549 and H1299 cells enhanced the PCNP level and transfection of PCNP short hairpin ribonucleic acid (shRNA) (sh-PCNP) reduced the expression of PCNP in A549 and H1299 cells (Fig. 2a). In addition, similar trends were observed in the levels of PCNP mRNA and protein (Fig. 2b–d). These results indicate that the models for PCNP overexpression and knockdown have been established successfully. PCNP overexpression promoted the proliferation and viability of A549 and H1299 cells, while PCNP knockdown showed reverse trends (Fig. 2e–g). Furthermore, PCNP overexpression increased the colony number and PCNP knockdown exhibited an opposite effect (Fig. 2h, i). In aggregate, the results suggest that PCNP can mediate the proliferation and viability of human lung adenocarcinoma cells.

### PCNP mediates the migration and invasion of human lung adenocarcinoma cells

**Fig. 3:**
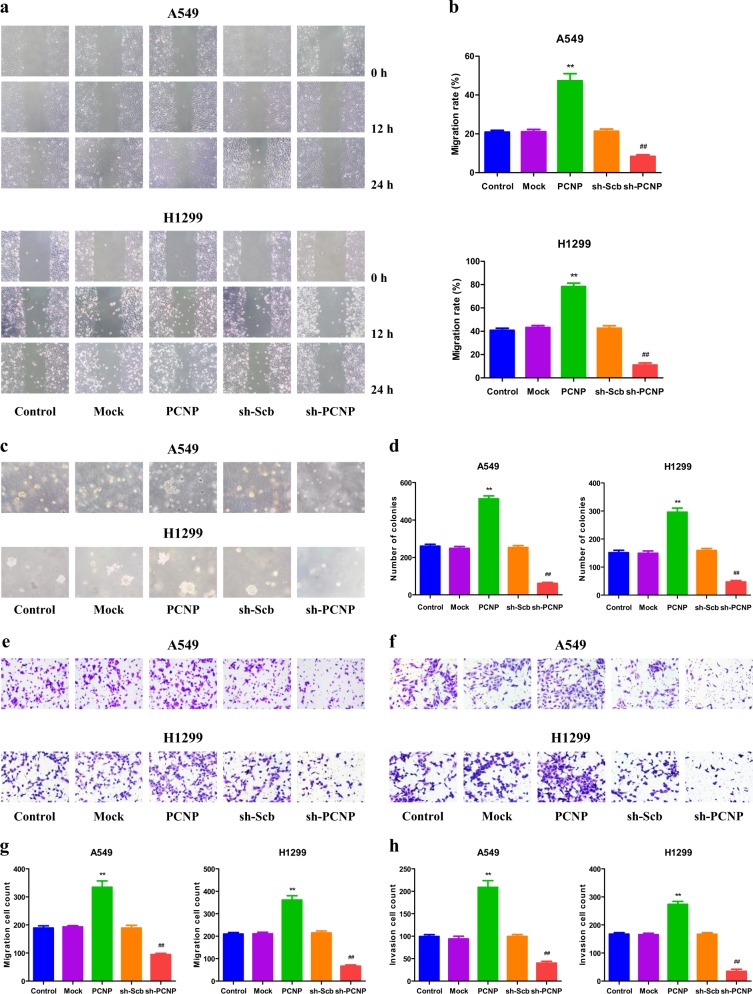
Effects of PCNP on the migration and invasion of human lung adenocarcinoma cells. **a** The effect of PCNP on cell migration was measured by wound healing assay; original magnification ×100. **b** The migration rates of A549 and H1299 cells were calculated. **c** Soft agar assay was performed to examine the anchorage-independent survival of cells; original magnification ×100. **d** The number of colonies was calculated. **e** Transwell assay was performed to assess the migration of A549 and H1299 cells; original magnification ×200. **f** Transwell assay was performed to assess the invasion of A549 and H1299 cells; original magnification ×200. **g** The numbers of the migrated cells were calculated. **h** The numbers of the invasive cells were calculated. Data are presented as mean ± SEM of three independent experiments; **P < 0.01 compared with the Mock group; ^##^*P* < 0.01 compared with the sh-Scb group

As shown in Fig. 3a, b, PCNP overexpression promoted the migration of A549 and H1299 cells and PCNP knockdown exhibited the opposite effect. Furthermore, PCNP overexpression increased the anchorage-independent growth of A549 and H1299 cells, while a reverse trend was observed in the sh-PCNP group (Fig. 3c, d). The migration and invasion capacities of A549 and H1299 cells were improved in the PCNP group, whereas the sh-PCNP group showed opposite effects (Fig. 3e–h). Collectively, the results demonstrate that PCNP is involved in the regulation of the migration and invasion of human lung adenocarcinoma cells.

### PCNP modulates apoptosis through the signal transducers and activators of transcription (STAT) 3/5 pathway in human lung adenocarcinoma cells

**Fig. 4:**
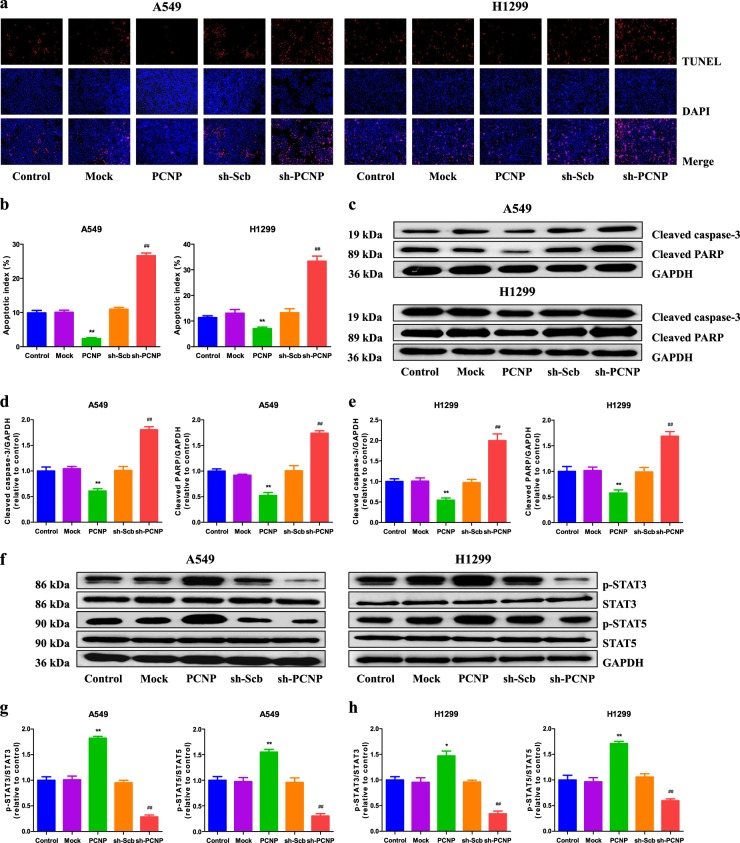
Effects of PCNP on the apoptosis and STAT3/5 signaling pathway in human lung adenocarcinoma cells. **a** The apoptotic levels of A549 and H1299 cells were measured by TdT-mediated dUTP-biotin nick end labeling (TUNEL) staining; original magnification ×100. **b** The percentages of TUNEL-positive cells were calculated. **c** Western blotting analysis for the expression of cleaved caspase-3 and cleaved PARP in A549 and H1299 cells. GAPDH was used as the loading control. **d**, **e** The densitometry analyses of cleaved caspase-3 and cleaved PARP were performed in A549 and H1299 cells, normalized to the corresponding GAPDH level. **f** Western blotting analysis for the expression of p-STAT3, STAT3, p-STAT5, and STAT5 in A549 and H1299 cells. GAPDH was used as the loading control. **g**, **h** The densitometry analyses of p-STAT3, STAT3, p-STAT5, and STAT5 were performed in A549 and H1299 cells, normalized to the corresponding GAPDH level. Data are presented as mean ± SEM of three independent experiments; *P < 0.05; **P < 0.01 compared with the Mock group; ^##^*P* < 0.01 compared with the sh-Scb group

The apoptotic index decreased in the PCNP group compared with the Mock group and increased in the sh-PCNP group compared with the sh-Scb group (Fig. 4a, b). Furthermore, similar trends were observed in the expression levels of cleaved caspase-3 and cleaved poly adenosine diphosphate-ribose polymerase (PARP) in human lung adenocarcinoma cells (Fig. 4c–e). Recent studies have demonstrated that STAT3 and STAT5 are persistently hyperactivated (phosphorylated) in different types of cancer^21–23^. The constitutive activation of STAT3 and STAT5 play vital roles in the proliferation, survival, apoptosis, and angiogenesis of cancer cells^21,24,25^. The protein levels of phospho (p)-STAT3 and p-STAT5 in the PCNP group were higher than those in the Mock group, whereas the two protein levels in the sh-PCNP group were lower than those in the sh-Scb group (Fig. 4f–h). Taken together, the results indicate that PCNP modulates apoptosis via STAT3/5 pathway in human lung adenocarcinoma cells.

### PCNP modulates autophagy via the phosphatidylinositol 3-kinase (PI3K)/Akt/mammalian target of rapamycin (mTOR) pathway in human lung adenocarcinoma cells

**Fig. 5:**
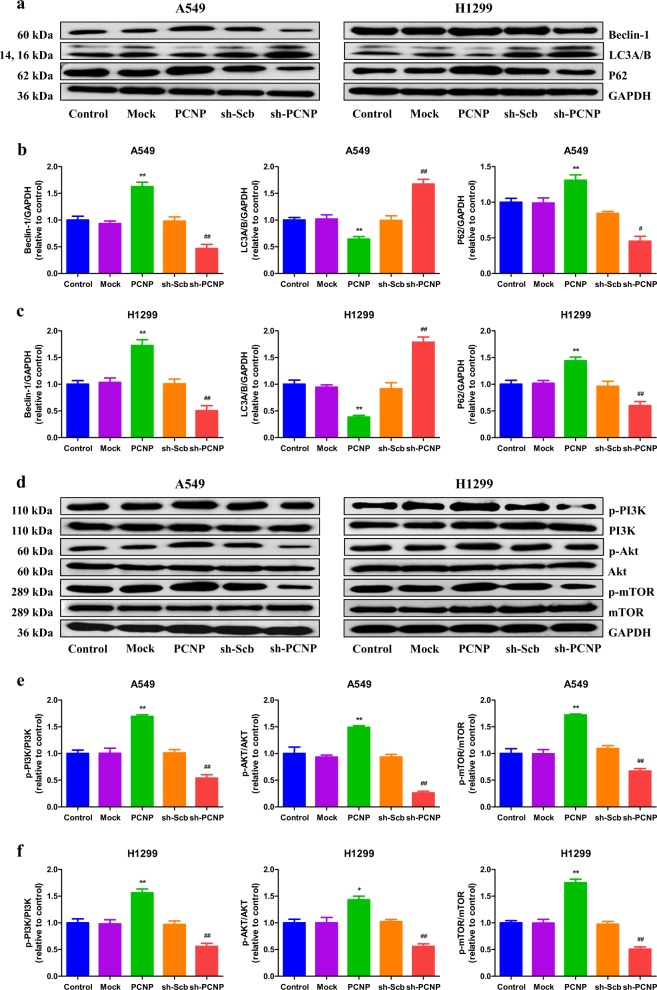
Effects of PCNP on the autophagy and PI3K/Akt/mTOR signaling pathway in human lung adenocarcinoma cells. **a** Western blotting analysis for the expression of Beclin-1, LC3A/B, and P62 in A549 and H1299 cells. GAPDH was used as the loading control. **b**, **c** The densitometry analyses of Beclin-1, LC3A/B, and P62 were performed in A549 and H1299 cells, normalized to the corresponding GAPDH level. **d** Western blotting analysis for the expression of p-PI3K, PI3K, p-Akt, Akt, p-mTOR, and mTOR in A549 and H1299 cells. GAPDH was used as the loading control. **e**, **f** The densitometry analyses of p-PI3K, PI3K, p-Akt, Akt, p-mTOR, and mTOR were performed in A549 and H1299 cells, normalized to the corresponding GAPDH level. Data are presented as mean ± SEM of three independent experiments; *P < 0.05; **P < 0.01 compared with the Mock group; ^#^*P* < 0.05; ^##^*P* < 0.01 compared with the sh-Scb group

Autophagy is a highly regulated process in which the constituents are delivered to lysosomes for degradation in cells^26^. Autophagy plays key roles in the development, physiology, and homeostasis. Dysregulated autophagy can lead to a number of pathophysiological conditions^27^. Autophagy is involved in the aetiology of cancer, playing the pro-survival or pro-death role depending on the types and stages of cancer^28^. An inefficient autophagy contributes to tumorigenesis and has been found in the early stage of cancer. However, increased autophagy plays an important role in advanced malignancies correlating with an metastatic/invasive phenotype^29,30^. Beclin 1, LC3, and p62 are involved in autophagy and have been widely regarded as autophagic markers^31,32^. Our results showed that PCNP overexpression increased the protein levels of Beclin 1 and P62, while PCNP knockdown decreased the expression levels of these proteins (Fig. 5a–c). In addition, the protein level of LC3A/B exhibited a reverse trend. An increasing number of studies have demonstrated that the PI3K/Akt/mTOR pathway plays a key role in regulating cell autophagy^33–35^. The results indicated that PCNP overexpression enhanced the protein levels of p-PI3K, p-Akt, and p-mTOR, whereas PCNP knockdown exerted opposite effects (Fig. 5d–f). These results together suggest that PCNP modulates autophagy via the PI3K/Akt/mTOR pathway in human lung adenocarcinoma cells.

### PCNP regulates the growth and angiogenesis of human lung adenocarcinoma xenograft tumors in nude mice

**Fig. 6:**
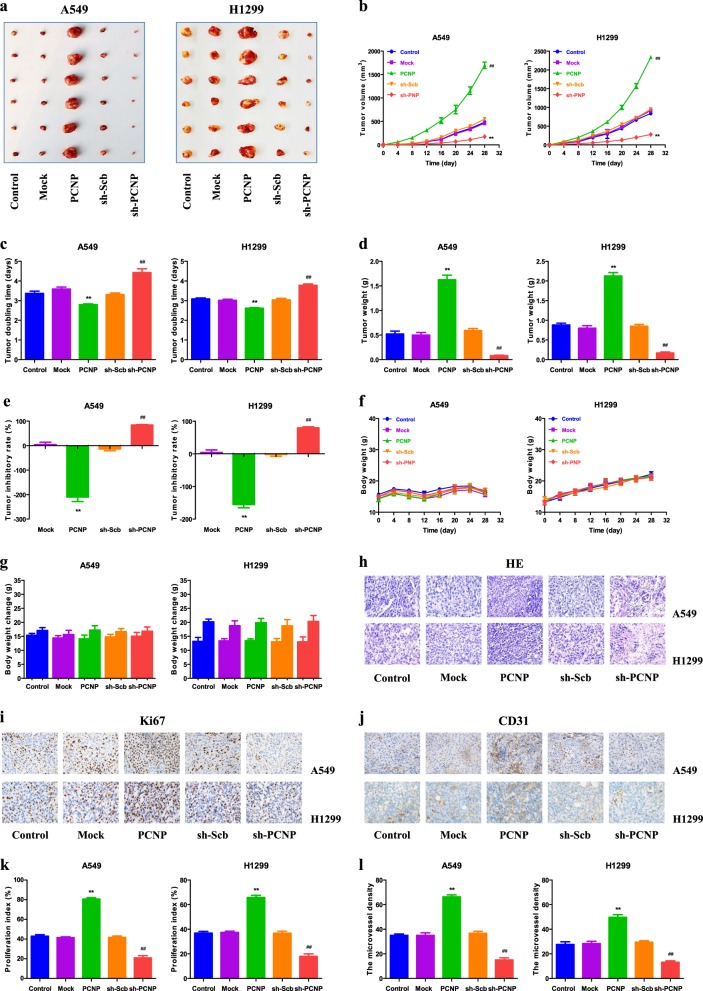
Effects of PCNP on the growth, proliferation index (PI), and MVD of A549 and H1299 xenograft tumors in nude mice. **a** Representative xenografts dissected from different groups of nude mice were shown. **b**, **c** The tumor volume of each group was measured every day and the tumor volume doubling time (TVDT) was calculated. **d**, **e** The tumors were weighed and the inhibition rates of tumor growth were calculated. **f**, **g** The body weight change curve of each group during the experiment and the body weight of each group on the first day (day 0) and the last day (day 28). **h**, **i**, **j** Representive photographs of hematoxylin and eosin (HE), Ki67, and cluster of differentiation 31 (CD31) staining in A549 and H1299 xenograft tumors; original magnification ×400. **k**, **l** The PI and MVD were calculated. Values are presented as mean ± SEM (*n* = 6); **P < 0.01 compared with the Mock group; ^##^*P* < 0.01 compared with the sh-Scb group

A549 and H1299 cells have been adopted to establish subcutaneous xenograft tumor models^36–38^. We further investigated the effects of PCNP on the growth of lung adenocarcinoma xenograft tumors. The results indicated that PCNP overexpression significantly promoted the growth of xenograft tumors when compared with the Mock group. Nevertheless, PCNP knockdown notably reduced the growth of xenograft tumors compared to the sh-Scb group (Fig. 6a–e). Furthermore, no significant difference was detected in body weight among groups (Fig. 6f, g). The results of Ki67 staining indicated that PCNP overexpression increased the in vivo proliferation of lung adenocarcinoma cells and a reverse effect was observed in the sh-PCNP group. Moreover, the microvessel density (MVD) of lung adenocarcinoma xenograft tumors exhibited a similar trend (Fig. 6h–l). In sum, these results reveal that PCNP mediates the growth and angiogenesis of human lung adenocarcinoma xenograft tumors.

## Discussion

Recent studies suggest that the mRNA expression of PCNP could be detected in several types of cancer, such as HepG2 hepatoma cells, HT-1080 fibrosarcoma cells, and U-937 myeloid leukemia cells, suggesting that PCNP may be involved in tumorigenesis^18,19^. In addition, our previous study has demonstrated that PCNP could be detected in human neuroblastoma cells both at mRNA and protein levels^20^. However, the expression level of PCNP in lung adenocarcinoma is still unknown. In this study, our results suggested that PCNP level was higher in lung adenocarcinoma tissue compared with adjacent non-tumor tissue. Furthermore, PCNP expression was associated with T classification of lung adenocarcinoma. In light of the results, we can conclude that PCNP level is high in lung cancer tissue but low in adjacent non-tumor tissue, indicating that PCNP is a novel promising biomarker for diagnosis and prognosis of lung cancer and may be involved in the procession of lung cancer.

Our previous study has demonstrated that PCNP can mediate the growth of human neuroblastoma cells via MAPK and PI3K/Akt/mTOR pathways^20^. Nevertheless, the mechanism of action of PCNP on the growth of lung adenocarcinoma has not been clarified. The human lung adenocarcinoma cell lines A549 and H1299 have been adopted to evaluate the therapeutic effects of different agents/drugs^36–38^. In this study, A549 and H1299 cells were used to detect the mechanism of action of PCNP on the growth of lung adenocarcinoma. It has been shown that PCNP overexpression could decrease the proliferation, viability, migration, and invasion of SH-SY5Y and SK-N-SH cells, while PCNP knockdown exhibits opposite effects^20^. In the present study, our data indicated that PCNP overexpression increased the proliferation and viability and promoted the migration and invasion of A549 and H1299 cells, while PCNP knockdown played opposite roles, suggesting that PCNP is involved in the procession of human lung adenocarcinoma cells. However, it should be noted that PCNP could exhibit different effects on human neuroblastoma cells and lung adenocarcinoma cells, which can be attributed to the heterogeneity of tumor types.

Apoptosis is a key regulatory process for the maintenance and development of homeostasis in multicellular organisms^39^. Two apoptotic pathways have been elucidated in mammals: mitochondria-mediated and death receptor-dependent pathways^40^. A variety of stimuli activate caspases and activated caspase-3 can further cleave PARP, resulting in the occurrence of apoptosis^41^. The results indicated that PCNP overexpression notably decreased the apoptotic index and expression levels of cleaved caspase-3 and PARP, while PCNP knockdown significantly increased apoptosis, indicating that PCNP mediates mitochondria-dependent apoptotic pathway in human lung adenocarcinoma cells. STAT proteins are transcription factors that regulate various biological processes^42^. STAT3 activation could promote the expression of gene products required for cell-cycle regulators, inhibitors of apoptosis, and inducers of angiogenesis, resulting in increased proliferation and decreased apoptosis in various cancer cells^43,44^. Furthermore, STAT5 also mediates proliferation and apoptosis in many cancer cells^43,45^. Recent studies have found that both STAT3 and STAT5 are activated in lung cancer cells and tissues, indicating functional roles for these proteins in lung cancer^21,46^. In line with these studies, our results suggested that the protein levels of p-STAT3 and p-STAT5 in the PCNP group were higher than those in the Mock group. However, the levels of these proteins were lower in the sh-PCNP group when compared with the sh-Scb group. Therefore, it can be concluded that PCNP can modulate apoptosis via STAT3/5 pathway in human lung adenocarcinoma cells.

Autophagy is an evolutionarily ancient and conserved catabolic process, in which cytoplasmic materials are sequestered in vesicles and delivered to lysosomes for degradation, resulting in the turnover of cell constituents and the generation of energy and macromolecular precursors^26,47^. Autophagy plays a multifactorial role in the initiation and procession of cancer, as well as in the effectiveness of therapeutic interventions against cancer^48^. It has been revealed that normal tissues are less autophagy-dependent than tumors. Thus, inhibition of autophagy can be a useful strategy in the treatment of cancer^49^. Our results showed that PCNP overexpression increased the autophagy level and PCNP knockdown exhibited opposite effects. Signals that activate the autophagic process typically originate from many conditions of stress, such as oxidative stress, hypoxia, starvation, endoplasmic reticulum stress, protein aggregation, and others^50,51^. Recent studies have shown that the PI3K/Akt/mTOR pathway is involved in the promotion of cell autophagy^34,52^. The results indicated that PCNP overexpression promoted the expression levels of p-PI3K, p-Akt, and p-mTOR, while PCNP knockdown reduced the levels of these proteins. The results suggest that PCNP modulates autophagy via PI3K/Akt/mTOR signaling pathway in human lung adenocarcinoma cells.

A549 and H1299 cells have been adopted to establish xenograft tumor models^36–38^. Then we detected the role of PCNP in the growth of lung adenocarcinoma xenograft tumors in nude mice. PCNP overexpression markedly promoted the growth of lung adenocarcinoma xenograft tumors, while PCNP knockdown notably reduced tumor growth. Ki67 is a nuclear non-histone protein expressed in proliferating cells^53^. Ki67 is considered a marker for cell proliferation and is widely used in detecting the proliferative level of cancer cells^20,53^. Similar to our findings in vitro, our results indicated that the Ki67 level was increased in the PCNP group but decreased in the sh-PCNP group. CD31, a specific biomarker for vascular endothelial cells (VEC), has been represented by the tumor MVD^20^. Our results suggested that PCNP overexpression promoted the level of CD31, whereas PCNP knockdown reduced the MVD in lung adenocarcinoma xenograft tumors, indicating that PCNP regulates the growth of human lung adenocarcinoma xenograft tumors through the mediation of angiogenesis.

In summary, our study indicates that PCNP could be detected in human lung adenocarcinoma cells. Importantly, the protein level of PCNP in human lung adenocarcinoma tissue is higher than that in adjacent non-tumor tissue. In addition, our findings reveal that PCNP mediates the proliferation, migration, and invasion of human lung adenocarcinoma cells via STAT3/5 and PI3K/Akt/mTOR signaling pathways. In light of its role in the development of human lung adenocarcinoma cells, PCNP may be considered as a promising biomarker for the diagnosis and prognosis in patients with lung adenocarcinoma. Furthermore, PCNP can be a potential therapeutic target and potent PCNP inhibitors can be designed and developed in the treatment of lung adenocarcinoma.

## Materials and methods

### Tissue samples

The expression levels of PCNP were detected in 63 human lung adenocarcinoma specimens and corresponding non-tumor lung tissues (National Human Genetic Resources Sharing Service Platform, Shanghai, China) by IHC. The informed consent was obtained from each subject after approval of the Ethics Committee of the First Affiliated Hospital of Henan University (20180517). Then four fresh specimens of patients who underwent surgery were collected to detect the protein levels of PCNP. Pathological classification and clinical staging were evaluated according to the American Joint Committee on Cancer criteria^54^.

### Immunohistochemical staining

Tissue sections were evaluated by two experienced pathologists independently. The results were semi-quantitatively scored according to the percentage of positively stained cells (0, 0%; 1, 1–25%; 2, 26–50%; 3, 51–75%; 4, 76%–100%) and the staining intensity (0, negative; 1, weak; 2, moderate; 3, strong). The two scores were combined to come up with a final PCNP expression score for each specimen. The score can be defined as follows: 0–3, low expression; 4–7, high expression^55^.

### Cell culture

Human lung adenocarcinoma A549 and H1299 cell lines were obtained from Nanjing Kebai Biological Technology Co., Ltd. (Nanjing, Jiangsu, China). A549 cells were incubated with DMEM medium containing 10% fetal bovine serum (FBS), 100 µg/ml streptomycin, and 100 U/ml penicillin. H1299 cells were incubated with RPMI1640 medium containing 10% FBS, 100 µg/ml streptomycin, and 100 U/ml penicillin. Cells were cultured at 37 ˚C in a humidified atmosphere composed of 5% CO_2_ and 95% air.

### Overexpression and knockdown of PCNP

Overexpression and knockdown of PCNP were performed in A549 and H1299 cells as previously described^20^. The localization of PCNP in cancer cells was observed with a fluorescent microscope (Eclipse Ti, Nikon, Melville, NY, USA).

### RT-PCR

Total RNA was extracted from cells using Trizol reagent (Invitrogen, Carlsbad, CA, USA) and then treated with DNase I (Roche, Indianapolis, IN, USA). The RT-PCR reaction was performed as previously described^20^.

### Cell viability and proliferation assays

Cell viability and proliferation assays were separately performed using a MTS kit (Promega, Madison, WI, USA) and a Cell-Light EdU Apollo 567 In vitro Imaging Kit (RiboBio, Guangzhou, Guangdong, China) according to the manufacturer’s instructions. Cell proliferation rate (%) = (EdU-positive cells)/(total number of cells) × 100^56^.

### Colony formation assay

Cells were cultured at a density of 6 × 10^2^ cells/well in 6-well plates. After cultivation for two weeks, the colonies were washed with phosphate-buffered saline (PBS) buffer and fixed using 1 ml methanol for 15 min at room temperature. 1 ml crystal violet was then added to each well and incubated at room temperature for 30 min. The plates were washed thoroughly with deionized water and dried in air at room temperature. Then the plates were scanned and the number of colonies was counted.

### Wound healing assay

Confluent cells were scratched using a sterile 200 μl pipette tip. The detached cells and debris were removed by washing with PBS. The migration distance was observed with an Olympus CKX41 microscope and determined using Image J software (National Institute for Health, Bethesda, MD, USA). The migration rate (MR) was calculated as previously described^57^.

### Soft agar assay

Soft agar assay was performed as previously described^20^. Colonies were observed and counted using an Olympus CKX41 microscope.

### Migration and invasion assays

Migration and invasion assays were performed as previously described^20^. The numbers of stained cells were counted under a Zeiss Axioskop 2 plus microscope (Carl Zeiss, Thornwood, NY, USA).

### TUNEL assay

TUNEL staining was assessed by an In Situ Cell Death Detection Kit (Beyotime Biotechnology, Shanghai, China) following the manufacturer’s instructions. The apoptotic cells were observed using a fluorescent microscope. The percentage of cells positive for TUNEL was calculated using Image J software.

### Western blotting

Total protein was extracted from A549 and H1299 cells. Western blotting was carried out to determine the protein levels. The primary antibodies including anti-STAT3, anti-STAT5, anti-p-STAT3 (Tyr705), anti-p-STAT5 (Try694), anti-PI3K, anti-p-PI3K (Tyr458/Tyr199), anti-Akt, anti-p-Akt (Ser473), anti-mTOR, anti-p-mTOR (Ser2448), anti-P62, anti-LC3A/B, and anti-Beclin-1 antibodies were obtained from CST (Danvers, MA, USA). Anti-PCNP antibody was obtained from Abcam (Cambridge, UK). Anti-cleaved PARP, anti-cleaved caspase-3, and anti-GAPDH antibodies were provided by ProteinTech (Chicago, IL, USA). Secondary antibody conjugated to horseradish peroxidase was obtained from CST. The immunodetection was then visualized by an enhanced chemiluminescence detection system (Thermo Fisher Scientific, Rockford, IL, USA). The band intensities were semi-quantified by Image J software. The expression of each protein was normalized to the expression level of GAPDH.

### Animal study

Animal studies were approved by the Committee of Medical Ethics and Welfare for Experimental Animals of Henan University School of Medicine (HUSOM-2017-190). Animal experiments were performed as previously described^20^. Thirty (*n* = 6 per group) BALB/C nude mice (4-week-old, male) were purchased from Beijing Vital River Laboratory Animal Technology Co., Ltd. (Certificate No. SCXK (Jing) 2011–0011, Beijing, China). A549 and H1299 cells (2 × 10^6^ cells in 200 μl PBS) with overexpression or knockdown of PCNP were inoculated subcutaneously into the right flanks of nude mice. The body weighs and tumor volumes were daily measured. The tumor volume was calculated according to the formula: volume = L × W^2^/2, where L is the longest dimension and W is the dimension perpendicular to L^58^. The TVDT was calculated as TVDT = log2/log(V2/V1) × (T − T_0_), where V1 and V2 indicate tumor volumes at two time points and (T − T_0_) represents the time interval^59^. Then mice were sacrificed and tumor masses were removed and weighted. The inhibition rate (IR) of tumor growth was calculated as IR = [(A – B)/A] × 100%, where A and B represent the average tumor weights of the control group and the treatment group respectively^20^.

### HE staining

Tumor sections were fixed in 10% neutral buffered formalin solution, embedded in paraffin, cut at 5 μm thickness and stained with HE. Then tumor tissues were photographed.

### Immunohistochemistry (IHC)

Tumor samples were stained with the anti-Ki67 monoclonal antibody (CST, Danvers, MA, USA). The PI was defined as the percentage of positively stained cells among the total number of cells^60^. CD31 is a biomarker for VEC, and the immunostaining density of CD31 is regarded as tumor MVD^61^. Tumor sections were stained with CD31 antibody (CST, Danvers, MA, USA) to detect tumor MVD. Stained vessels with a clearly defined lumen or well-defined linear vessel shape were counted.

### Statistical analysis

Data are presented as mean ± SEM. The differences between multiple groups were analyzed by one-way analysis of variance using SPSS 17.0 software, followed by Tukey’s test. A *P* value of less than 0.05 was considered to be statistically significant.
